# A clinical study on the augmented therapeutic effects of transcutaneous auricular vagus nerve stimulation combined with repetitive transcranial magnetic stimulation in the treatment of moderate-to-severe depression

**DOI:** 10.12669/pjms.42.3.13028

**Published:** 2026-03

**Authors:** Shuhui Gao, Yuan Zhang, Kun Mi, Haizhu Yu, Mengxue Wu, Lin Li

**Affiliations:** 1 Shuhui Gao, Department of Physical Therapy, Hebei Provincial Mental Health Center, The Sixth People’s Hospital of Hebei Province, Baoding 071000, Hebei, China; 2 Yuan Zhang, Department of Physical Therapy, Hebei Provincial Mental Health Center, The Sixth People’s Hospital of Hebei Province, Baoding 071000, Hebei, China; 3 Kun Mi, Department of Physical Therapy, Hebei Provincial Mental Health Center, The Sixth People’s Hospital of Hebei Province, Baoding 071000, Hebei, China; 4 Haizhu Yu, Department of Physical Therapy, Hebei Provincial Mental Health Center, The Sixth People’s Hospital of Hebei Province, Baoding 071000, Hebei, China; 5 Mengxue Wu, Department of Physical Therapy, Hebei Provincial Mental Health Center, The Sixth People’s Hospital of Hebei Province, Baoding 071000, Hebei, China; 6 Lin Li, Department of Physical Therapy, Hebei Provincial Mental Health Center, The Sixth People’s Hospital of Hebei Province, Baoding 071000, Hebei, China

**Keywords:** Moderate-to-severe depression, Repetitive transcranial magnetic stimulation, Transcutaneous ear acupoint, Treatment, Vagus nerve stimulation

## Abstract

**Objective:**

To evaluate the clinical efficacy of transcutaneous auricular vagus nerve stimulation (taVNS) combined with repetitive transcranial magnetic stimulation (rTMS) in the treatment of moderate-to-severe depression.

**Methodology:**

This was a retrospective study. A total of 180 patients diagnosed with moderate-to-severe depression and admitted to Hebei Provincial Mental Health Center between July 2023 to March 2025 were enrolled and randomized into three groups(*n=* 60 each) using a random number table: the taVNS group (Group-A), the rTMS group (Group-R) and the taVNS + rTMS group (Group-C). All patients underwent a 4-week treatment course. Clinical outcomes, changes in depression severity, sleep quality and incidence of adverse events were assessed post-treatment. All outcome assessments were conducted under blinded conditions using standardized rating scales.

**Results:**

The overall response rate was 85% in Group-C, significantly higher than the 70% in Group-A and the 66% in Group-R (*p=* 0.02). Additionally, patients in Group-C showed significantly greater reductions in both Clinical Global Impression and Pittsburgh Sleep Quality Index scores compared with Groups A and R (both *p=* 0.00). No statistically significant differences were observed among the three groups in the incidence of adverse events(*p=* 0.60).

**Conclusion:**

The combination of taVNS and rTMS demonstrates a synergistic effect in the treatment of moderate-to-severe depression, which is considered a safe and effective therapeutic strategy as it can significantly improve clinical symptoms and sleep quality without increasing adverse effects.

## INTRODUCTION

Depression is a common, chronic, recurrent psychiatric disorder characterized by a high prevalence, relapse rate, suicide risk, disability rate and substantial disease burden. Its core symptom is a persistently low mood or emotional state, often accompanied by varying degrees of cognitive and behavioral disturbances. Cognitive impairments in depression may include deficits in attention, judgment, executive functioning and memory, which are primarily attributed to dysfunctions in the brain’s processes for acquiring, storing, integrating and processing information.^1^ Somatic symptoms, such as pain, fatigue, autonomic dysfunction and central nervous system disturbances, are also prevalent, with approximately 70% of patients with depression experiencing such symptoms.^2^ Current treatment strategies for depression rely predominantly on pharmacotherapy and psychotherapy.

However, clinical observations suggest that approximately 30% of patients with moderate-to-severe depression show inadequate responses to at least two antidepressants with different chemical structures, a condition commonly referred to as treatment-resistant depression (TRD).^3^ As a result, the development of effective non-pharmacological interventions for TRD has become a key focus in depression research. Invasive vagus nerve stimulation (VNS) has been approved for the treatment of epilepsy, depression, obesity and post-stroke rehabilitation. However, this method requires surgical implantation, is associated with adverse effects and remains costly.^4^ In contrast, transcutaneous auricular vagus nerve stimulation (taVNS) is a novel, non-invasive approach that stimulates the auricular branch of the vagus nerve through the ear acupoints. By modulating emotion-related brain regions, taVNS has demonstrated antidepressant effects and is increasingly applied in the clinical treatment of neurological, psychiatric and behavioral disorders.^5^

Repetitive transcranial magnetic stimulation (rTMS) is a non-invasive, painless neuromodulation technique widely used in neuropsychiatric practice. Numerous studies have confirmed the antidepressant effect of high-frequency rTMS^6^. Sheth SA et al.^7^ proposed that rTMS may enhance the efficacy of antidepressant medications through synergistic mechanisms, while also avoiding the adverse effects commonly associated with pharmacological treatments. Based on these findings, we conducted a clinical study to investigate the augmented therapeutic effects of taVNS combined with rTMS in patients with moderate-to-severe depression.

## METHODOLOGY

This was a retrospective study. A total of 180 patients diagnosed with moderate-to-severe depression and treated at Hebei Provincial Mental Health Center between July 2023 to March 2025 were enrolled in this study. Using a random number table, the participants were randomized into three groups (*n=* 60 each): the taVNS group (Group-A), the rTMS group (Group-R) and the taVNS + rTMS group (Group-C). In Group-A, there were 22 males and 38 females, aged 22-54 years, with a mean age of 45.12 ± 8.26 years. Group-R comprised 26 males and 34 females, aged 25-55 years, with a mean age of 45.58 ± 9.03 years. Group-C included 25 males and 35 females, aged 23-54 years, with a mean age of 45.30 ± 8.43 years. No significant differences were observed among the three groups in baseline characteristics, indicating comparability (Table-I).

**Table-I:** Baseline characteristics of the three groups (*x̅*±*s*, n = 60 per group).

Item	Group-A	Group-R	Group-C	F-value	P-value
Age (years)	45.12±8.26	45.58±9.03	45.30±8.43	0.18	0.86
Male (n)	22	26	25	0.31	0.58
Disease duration (months)	36.85±12.63	36.52±11.71	35.89±10.37	0.31	0.75
Baseline HAMD score	23.08±4.41	23.12±4.06	23.30±4.86	0.22	0.83
Years of education	15.47±8.46	15.82±8.71	15.75±7.83	0.05	0.96

P > 0.05.

### Ethical approval:

The study was approved by the Institutional Ethics Committee of Hebei Provincial Mental Health Center (No.202326; Dated: July 21, 2023) and written informed consent was obtained from all participants.

### Inclusion criteria:

- Diagnosis of depression in accordance with the International Classification of Diseases (ICD) criteria.^8^
- Aged between 18 and 70 years.
- A score >17 on the 17-item Hamilton Depression Rating Scale (HAMD-17).
- No antidepressant treatment within the past two months, or discontinuation of antidepressants for more than four weeks.
- Poor response to at least two antidepressant medications of different pharmacological classes, administered at adequate doses and durations.
- No history of alcohol or substance abuse, or traumatic brain injury.
- No neurological or severe systemic physical illness and able to comply with study procedures.
- Provision of written informed consent by both the patient and their family.
- Availability of complete clinical data.

### Exclusion criteria:

- Neurological disorders caused by other identifiable etiologies.
- Severe dysfunction of the heart, liver, or kidneys.
- Severe physical illness affecting mood or sleep.
- High risk of suicide.
- Pregnancy or lactation.

All patients received monotherapy with a single antidepressant. For those experiencing severe anxiety, agitation, or insomnia, short-term use of non-benzodiazepine sedatives was permitted. Patients in Group-A received taVNS using 0.25 × 13 mm acupuncture needles manufactured by Zhongyan Taihe. Stimulation was administered once daily, 20 minutes per session, five times per week. The stimulation sites were the bilateral auricular conchae, corresponding to the auricular area of the vagus nerve. Patients in Group-R were treated using the YIRUIDE rTMS stimulator with a figure-eight coil. The stimulation targeted the left dorsolateral prefrontal cortex. Stimulation parameters were set as follows: 100% of resting motor threshold, 60% stimulus intensity, 10 Hz frequency, 0.5 seconds of stimulation per train, five pulses per train, three-second inter-train interval, 300 repetitions per session, totaling 1500 pulses per day. Sessions were conducted once daily, five times per week. Patients in Group-C received both taVNS and rTMS as described above. All patients underwent a total of four weeks of treatment. Upon completion of the treatment period, all participants were evaluated using standardized rating scales. Assessments were performed under blinded conditions by trained raters.

### Outcome measures:

### Clinical Efficacy:

It was assessed using the reduction rate of the Hamilton Depression Rating Scale (HAMD-17). A reduction rate >75% was defined as significant response (SR), 25%-75% as partial response (PR) and <25% as no response. The overall response rate (ORR) was calculated as: ORR = (Number of SR + PR cases) / Total number of cases × 100%. The reduction rate of HAMD scores was calculated as follows: Reduction rate = (Pre-treatment HAMD score − post-treatment HAMD score) / Pre-treatment HAMD score × 100%. Severity of Illness Before and After Treatment: The Clinical Global Impression (CGI) scale was used to assess the severity of illness before and after treatment in each group. The CGI scale uses an 8-point scoring system ranging from 0 to 7, where: 0 = not assessed; 1 = normal, not at all ill; 2 = borderline mentally ill; 3 = mildly ill; 4 = moderately ill; 5 = markedly ill; 6= severely ill; and 7 = extremely ill.^9^ Sleep Quality: Sleep quality was evaluated using the Pittsburgh Sleep Quality Index (PSQI).^10^ Participants completed the questionnaire in approximately 5-10 minutes. The total score ranges from 0 to 21, with higher scores indicating poorer sleep quality. Adverse Events: Adverse events were recorded throughout the treatment period and included symptoms such as dizziness, headache, dry or itchy skin, nausea and vomiting.

### Statistical analysis:

All data were analyzed using SPSS 20.0. Continuous variables were expressed as mean ± standard deviation (*χ̅±s*). According to the data of each indicator in the pre-survey, the sample size is estimated by 95% confidence interval, and the largest one is the sample size of the study. The sample size required for each group was ≥90 cases on the basis of Fisher exact probability. The confidence interval is 95%, categorical data were presented as absolute numbers(*n*) or percentages(%). One-way analysis of variance was used to compare differences among groups and paired t-tests were applied for within-Group-Comparisons before and after treatment. The chi-square (χ²) test was used to compare proportions. A *p*-value of <0.05 was considered statistically significant.

## RESULTS

The ORR was 85% in Group-C, 70% in Group-A and 66% in Group-R. The ORR in Group-C was significantly higher than that in either monotherapy group (*p =* 0.02) (Table-II).

**Table-II:** Comparison of clinical efficacy among the three groups (*x̅*±*s*, n = 60 per group).

Group	SR	PR	NR	ORR*
Group-A	22	21	17	42(70%)
Group-R	27	18	15	40(66%)
Group-C	34	20	16	51(85%)
*χ* ^2^				5.16
P-value				0.02

* p < 0.05.

There were no significant differences in baseline CGI scores among the three groups (*p =* 0.84). Post-treatment CGI scores significantly decreased in all groups compared with baseline (*p =* 0.00). No significant difference was observed between Groups A and R in CGI score reduction (*p =* 0.81). In contrast, Group-C showed a significantly greater reduction in CGI scores compared with both A and R (*p =* 0.00) (Table-III).

**Table-III:** Comparison of CGI scores before and after treatment (*x̅*±*s*, n = 60 per group).

Group	Pre-treatment	Post-treatment*	t-value	P-value
Group-A*	5.03±1.06	3.01±0.55▲	13.16	0.00
Group-R*	4.98±0.87	3.04±0.79▲	12.79	0.00
Group-C*	5.01±0.80	1.65±0.56	25.08	0.00
F-value	0.20	13.42		
P-value	0.84	0.00		

* p < 0.05. Note: ▲t = 0.24, p = 0.81.

No significant differences were found in baseline PSQI scores among the three groups (*p =* 0.39). After treatment, all groups showed significantly reduced PSQI scores (*p =* 0.00); no significant difference was observed between Groups A and R (*p =* 0.57), while Group-C exhibited significantly greater improvement in sleep quality compared with the other two groups (*p =* 0.00) (Table-IV). The incidence of adverse events was 12% in Group-A, 10% in Group-R and 15% in Group-C. No statistically significant differences were observed in the incidence of adverse events among the three groups (*p =* 0.60) (Table-V).

**Table-IV:** Comparison of PSQI scores before and after treatment (*x̅*±*s*, n = 60 per group).

Group	Pre-treatment	Post-treatment*	t-value	P-value
Group-A*	14.60±2.33	8.47±1.35▲	17.60	0.00
Group-R*	14.63±2.27	8.64±1.71▲	16.32	0.00
Group-C*	15.01±2.82	6.73±2.26*	22.54	0.00
F-value	0.87	4.48		
P-value	0.39	0.00		

* p < 0.05. Note: ▲t = 0.53, p = 0.57.

**Table-V:** Comparison of adverse events (*x̅*±*s*, n = 60 per group).

Group	Dizziness	Headache	Dry/Itchy skin	Nausea and vomiting	Overall complication rate
Group-A	0	2	2	4	7(12%)
Group-R	1	3	2	0	6(10%)
Group-C	3	2	0	4	9(15%)
*χ* ^2^					0.29
P-value					0.60

p > 0.05.

## DISCUSSION

Our study showed that the ORR in Group-C was 85%, significantly higher than in Group-A (70%) and Group-R (66%) (*p =* 0.02). Furthermore, Group-C demonstrated significantly lower CGI and PSQI scores compared with Groups A and R (*p =* 0.00), suggesting that the combination of taVNS and rTMS enhances therapeutic efficacy and improves sleep quality in patients with TRD. Studies have shown that rTMS is effective in improving core depressive symptoms such as low mood, anhedonia and cognitive impairment.^11^ Given these benefits, rTMS is considered to have synergistic potential when combined with other therapeutic modalities, consistent with our findings. Gerges ANH et al.^12^ have emphasized the critical role of supraspinal neural pathways in the treatment of depression. These pathways involve several key nuclei, including the nucleus of the vagus nerve, the paraventricular nucleus of the hypothalamus, the locus coeruleus and the paratrigeminal nucleus. The stimulation of the auricular conchae activates afferent vagal fibers, which in turn engage this neural network, transmitting signals to the spinal cord and cerebral cortex. This mechanism may underlie the therapeutic effects of auricular acupuncture in modulating the pathological basis of depression. Additionally, VNS may act directly on supraspinal neural centers and thus improve the symptoms of psychiatric disorders, further supporting the clinical relevance of stimulating these pathways. Importantly, our study also found no statistically significant differences in the incidence of adverse events among the three groups(*p =* 0.60), indicating that the combined use of taVNS and rTMS does not increase the risk of treatment-related side effects.

Clinically, depressive disorders are classified into mild, moderate and severe categories based on the number, type and severity of symptoms. While hallmark symptoms include depressed mood, anhedonia and fatigue, early signs may also manifest as cognitive slowing, delayed reactions and poor memory, though these vary significantly between individuals.^13^ Although depression is not contagious, it is closely associated with stressful life events, pessimistic personality traits, a personal or family history of psychiatric illness, chronic physical conditions, alcohol abuse and substance misuse.^14^ Epidemiological data indicate that approximately 4.4% of the global population is affected by depression, making it one of the leading causes of disability worldwide.^15^ Current treatment strategies primarily include pharmacotherapy and psychotherapy. Antidepressants remain the cornerstone of treatment, typically taking effect within 2-4 weeks. Psychotherapy can also yield therapeutic benefits. However, the onset of action for antidepressants is relatively slow and both pharmacological and psychotherapeutic interventions alone often yield suboptimal outcomes in patients with moderate-to-severe depression. This highlights the significant importance of identifying more effective and timely therapeutic approaches.

Since 2005, VNS has been applied in the treatment of depression and its antidepressant effects are now widely recognized.^16^ The vagus nerve is the longest and most extensively distributed cranial nerve in the human body, exerting regulatory effects on multiple organ systems and playing a vital role in maintaining homeostasis. Traditional VNS involves surgically implanting direct current electrodes at the carotid bifurcation to stimulate both the vagus and sympathetic nerves. It has been approved for the treatment of refractory epilepsy and chronic TRD and it has also been investigated in conditions such as bipolar disorder, asthma, Alzheimer’s disease, traumatic brain injury and cerebral infarction.^17^ However, traditional VNS is an invasive surgical procedure, which limits its clinical application due to surgical risks, technical complexity, potential side effects and high costs. Anatomically, the auricular concha is the only external site where the vagus nerve is accessible on the body surface. Stimulation of the concha zone has been shown to modulate vagal pathways within the brainstem and central nervous system. By integrating the traditional Chinese medicine (TCM) theory of auricular acupoints with modern neuroscience, stimulation of the auricular branch of the vagus nerve via acupuncture can induce regulatory effects similar to those of invasive VNS.^4^

The taVNS technique offers several advantages. It is non-invasive, easy to administer, cost-effective and associated with minimal side effects. Studies have demonstrated that acupuncture at the auricular conchae can directly activate afferent vagal fibers,^18^ providing a novel approach for treating psychiatric disorders within the TCM framework. Research^19^ further showed that between 40% and 90% of patients with depression suffer from comorbid chronic insomnia. Sleep disturbances often persist even after mood symptoms have improved and are considered one of the most common residual symptoms of depression, significantly influencing patient prognosis. Notably, traditional VNS alone has shown limited efficacy in improving insomnia.

The rTMS modality is a non-invasive, safe and technically accessible neurophysiological intervention. It produces antidepressant effects by modulating mood-related neural circuits and reducing the sensitivity of the hypothalamic-pituitary-adrenal axis. The underlying mechanism involves rTMS using a magnetic field to induce depolarization of neuronal membranes beneath the coil, thereby altering neural circuit activity and achieving non-invasive modulation of brain function.

### Limitations:

First, the retrospective design limits causal inference, though we employed rigorous statistical controls. Second, the moderate sample size may affect generalizability, though power analysis suggested adequate power for primary outcomes. Fourth, while we standardized assessment tools, some variability in intervention delivery between nurses may have occurred despite training. Finally, While this retrospective design utilized prospectively collected standardized assessments, the lack of randomization and potential variability in documentation practices may introduce bias compared to prospective studies.

## CONCLUSIONS

For patients with moderate-to-severe depression, the combined use of taVNS and rTMS yields superior clinical outcomes. This combination can alleviate the symptoms of TRD and improve sleep quality without increasing the incidence of adverse events. These findings suggest that taVNS combined with rTMS represents a promising therapeutic option for the treatment of depression.

### Authors’ Contributions:

**SG, YZ** and **KM:** Performed the studies, data collection and drafted the manuscript and are responsible and accountable for the accuracy or integrity of the work.

**HY, MW** and **LL:** Literature search, performed the statistical analysis and participated in its design.

All authors have read and approved the final manuscript.
